# Effects of metatarsal domes on plantar pressures in older people with a history of forefoot pain

**DOI:** 10.1186/s13047-020-00388-x

**Published:** 2020-05-06

**Authors:** Karl B. Landorf, Claire A. Ackland, Daniel R. Bonanno, Hylton B. Menz, Saeed Forghany

**Affiliations:** 1 Discipline of Podiatry, School of Allied Health, Human Services and Sport, La Trobe University, Melbourne, Victoria 3086 Australia; 2 La Trobe Sport and Exercise Medicine Research Centre, La Trobe University, Melbourne, Victoria 3086 Australia; 3 Musculoskeletal Research Center, School of Rehabilitation Sciences, Isfahan University of Medical Sciences, Isfahan, Iran; 4 Centre for Health Sciences Research, University of Salford, Salford, M5 4WT England

**Keywords:** Aged, Pain, Forefoot, human, Orthoses, Orthotic devices, Biomechanics, Kinetics, Plantar pressure

## Abstract

**Background:**

Forefoot pads such as metatarsal domes are commonly used in clinical practice for the treatment of pressure-related forefoot pain, however evidence for their effects is inconsistent. This study aimed to evaluate the effects on plantar pressures of metatarsal domes in different positions relative to the metatarsal heads.

**Methods:**

Participants in this study included 36 community-dwelling adults aged 65 or older with a history of forefoot pain. Standardised footwear was used and plantar pressures were measured using the pedar®-X in-shoe plantar pressure measurement system. Peak pressure, maximum force and contact area were analysed using an anatomically-based masking protocol that included three forefoot mask sub-areas (proximal to, beneath, and distal to the metatarsal heads). Data were collected for two different types of prefabricated metatarsal domes of different densities (*Emsold metatarsal dome* and *Langer PPT metatarsal pad*) in three different positions relative to the metatarsal heads. Seven conditions were tested in this study: (i) control (no pad) condition, (ii) Emsold metatarsal dome positioned 5 mm proximal to the metatarsal heads, (iii) Emsold metatarsal dome positioned in-line with the metatarsal heads, (iv), Emsold metatarsal dome positioned 5 mm distal to the metatarsal heads, (v) Langer PPT metatarsal pad positioned 5 mm proximal to the metatarsal heads, (vi) Langer PPT metatarsal pad positioned in-line with the metatarsal heads, and (vii) Langer PPT metatarsal pad positioned 5 mm distal to the metatarsal heads.

**Results:**

When analysed with the mask that was distal to the metatarsal heads, where the plantar pressure readings were at their highest, all metatarsal dome conditions led to significant reductions in plantar pressure at the forefoot compared to the control (no pad) condition (F_3.9, 135.6_ = 8.125, *p* < 0.001). The reductions in plantar pressure were in the order of 45–60 kPa. Both the Emsold metatarsal dome and the Langer PPT metatarsal pad, when positioned proximal to the metatarsal heads, managed to achieve this without adversely increasing plantar pressure proximally where the pad was positioned, however the Emsold metatarsal dome was most effective.

**Conclusions:**

Metatarsal domes reduce plantar pressure in the forefoot in older people with a history of forefoot pain. All metatarsal dome conditions significantly reduced peak pressure in the forefoot, however metatarsal domes that were positioned 5 mm proximal to the metatarsal heads provided the best balance of reducing plantar pressure distal to the metatarsal heads, where the pressure is at its greatest, but not adversely increasing plantar pressure proximally, where the bulk of the pad is positioned. In this proximal position, the Emsold metatarsal dome was more effective than the Langer PPT metatarsal pad and we cautiously recommend this forefoot pad for alleviating forefoot pressure in older people with forefoot pain.

## Introduction

Foot pain is a common complaint in older people – it is estimated that between 20 and 29% of older people have foot pain [1–3]. Further, foot pain in older people can affect mobility, gait and balance [4, 5]. One region of the foot that is commonly affected by pain is the forefoot [6], which is defined by pain in the region of the metatarsals heads [7, 8]. In older people, forefoot pain is the most common type of foot pain, accounting for approximately 37% of all foot pain [6].

Forefoot pain in older people is associated with many causes, including high plantar pressures under the forefoot [4, 9]. Therefore, redistribution of high forefoot plantar pressures using forefoot pads may reduce forefoot pain [10]. There are many different types of forefoot pads, although a commonly used type is the *metatarsal dome*, a teardrop shaped pad that is usually positioned just proximal to the middle metatarsal heads [11]; although, the exact position of metatarsal domes has not been clearly defined and evaluated.

We previously tested the plantar pressure redistribution properties of forefoot pads, including a metatarsal dome that was tested in two positions (10 mm proximal and 5 mm distal to the metatarsal heads) [12]. We found metatarsal domes in both positions reduced forefoot peak pressure (9% reduction with the proximal dome and 17% reduction with the distal dome), however we could not accurately determine where in the forefoot peak pressure was reduced, nor could we adequately explain how the metatarsal domes achieved their effect.

Currently, there is limited data on the effect of metatarsal domes on forefoot plantar pressures. Further, the optimal position of a metatarsal dome for redistributing plantar pressure is currently unknown [13–15]. The aim of this study was to measure the effect of different metatarsal domes in different positions on plantar pressures in older people with a history of forefoot pain.

## Methods

### Ethics approval

Ethics approval was obtained from the La Trobe University Faculty Human Ethics Committee – application FHEC12/207. All participants signed written informed consent prior to recruitment into the study.

### Participants

The participants were 36 community-dwelling older adults from Melbourne, Australia.

#### Inclusion criteria

Participants were eligible if they:

(i) were aged 65 years or older;
(ii) were community-dwelling;
(iii) had forefoot pain or a previous history of forefoot pain;
(iv) were able to walk household distances (10 m) without the use of a walking aid;
(v) were cognitively aware, so they could understand the requirements of the project;
(vi) were able to speak basic English, so they could provide informed consent prior to participation, follow instructions during the project, and to answer research questions accurately.

#### Exclusion criteria

Participants were excluded from the study if they:

(i) had any self-reported condition that may have affected lower limb sensation or muscle strength such as a stroke, polio, diabetic peripheral neuropathy;
(ii) had any lower limb surgery in the previous 3 months;
(iii) had any lower limb amputations that may affect lower limb function.

#### Recruitment

All participants were recruited from a study population involved in a previous clinical trial [16, 17]. Participants in this study were only recruited after they had completed all requirements in the clinical trial.

#### Sample size determination

The sample size was determined prior to conducting the study using an appropriate formula [18]. A sample size of 36 provides an 80% probability of detecting a clinically meaningful difference between interventions of 60 kPa in peak plantar pressure. The standard deviation used to determine this sample size was taken from a similar study that measured plantar pressures in older people [19] and was set at 90 kPa. The alpha level was set at 0.05.

### Setting

The study was performed in a research room in the Health Sciences Clinic at La Trobe University in Melbourne, Australia.

### Interventions

Two different brands of prefabricated metatarsal domes (i.e. pads) were used: (i) *Emsold metatarsal dome* (Emsold-Gesellschaft Gert Helmers GmbH & Co. KG, Rastede, Germany), and (ii) *Langer PPT metatarsal pad* (Langer Biomechanics, Ronkonkoma, New York, USA). Each dome differs in its hardness; the Emsold metatarsal dome has an average Shore A hardness of 11 durometer, while the Langer PPT metatarsal pad is harder, with an average Shore A hardness of 20 durometer. Both metatarsal domes are 6 mm at their highest point and are teardrop shaped. The domes were dispensed in two different sizes depending on foot size. The Emsold metatarsal dome was supplied in sizes 3 and size 5, which correspond to the Langer PPT metatarsal pad sizes small and medium, respectively. The metatarsal domes were supplied free of charge by Briggate Medical Company (Braeside, Victoria, Australia).

**Fig. 1:**
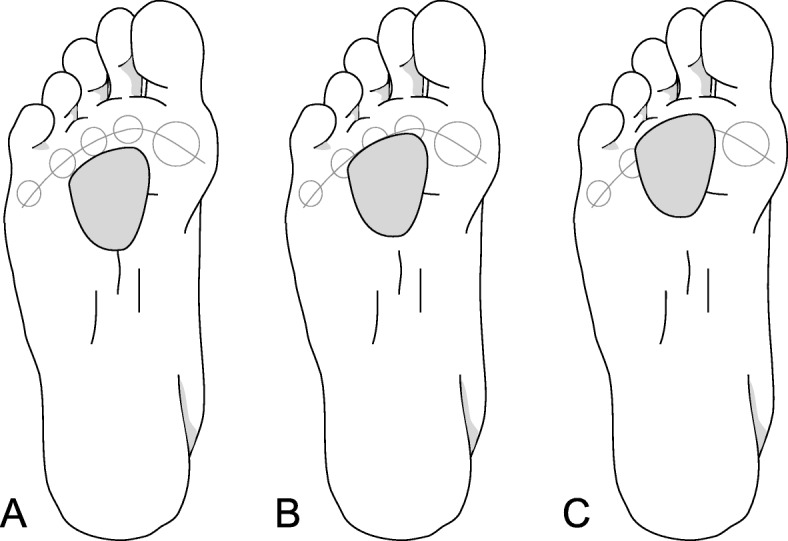
The three positions that the metatarsal domes were evaluated in (**a** = proximal, **b** = in-line, **c** = distal)

The metatarsal domes were adhered using double-sided adhesive tape to a cardboard template (similar to an insole) sized to fit into the shoe – this prevented the pad from moving during testing. The template was positioned between the plantar surface of the foot and the bottom of the inside of the shoe. All participants were tested in a control shoe (see protocol below), so the only difference between conditions was the type of metatarsal dome (with the control condition having no dome). Each dome was tested in three positions relative to the metatarsal heads: a proximal position, an in-line position, and a distal position (Fig. 1).

Therefore, there was one control condition and six different metatarsal dome conditions assessed (i.e. seven conditions in total):

(i) shoe with cardboard template only (no pad control);
(ii) Emsold metatarsal dome positioned 5 mm proximal to the metatarsal heads;
(iii) Emsold metatarsal dome positioned in-line with the metatarsal heads;
(iv) Emsold metatarsal dome positioned 5 mm distal to the metatarsal heads;
(v) Langer PPT metatarsal pad positioned 5 mm proximal to the metatarsal heads;
(vi) Langer PPT metatarsal pad positioned in-line with the metatarsal heads;
(vii) Langer PPT metatarsal pad positioned 5 mm distal to the metatarsal heads.

The borders of the metatarsal dome conditions in relation to the anatomical landmarks of the foot are shown below in Table 1.

**Table 1:** Positioning and approximate borders of the metatarsal domes

Position tested*	Proximal border	Distal border	Medial border	Lateral border
**5 mm proximal to the metatarsal heads**	15 mm distal to the styloid process of the 5th metatarsal	5 mm proximal to the metatarsal heads	Medial margin of the 2nd metatarsal	Lateral margin of the 4th metatarsal
**In-line with the metatarsal heads**	20 mm distal to the styloid process of the 5th metatarsal	In-line with the metatarsal heads	Medial margin of the 2nd metatarsal	Lateral margin of the 4th metatarsal
**5 mm distal to the metatarsal heads**	25 mm distal to the styloid process of the 5th metatarsal	5 mm distal to the metatarsal heads	Medial margin of the 2nd metatarsal	Lateral margin of the 4th metatarsal

*Notes: (i) both types of metatarsal domes (Emsold and Langer) were tested in all three positions, and (ii) for full details of how the metatarsal heads were located and marked on the template see the ‘Protocol’ sub-section in the Methods

#### Randomisation

To minimise ordering effects associated with the administration of the conditions, the order of testing for each of the domes was randomised according to a random computer-generated sequence using Microsoft Excel® 2007 (Microsoft, Redmond, WA, USA).

### Blinding

The participants were blinded to the types of interventions used. They were issued a standard set of instructions informing them that they were being tested with different forefoot pads but the designs and materials used were not revealed to them. Assessor blinding was not carried out due to the difficulty in concealing the interventions. However, because the outcome measures were objective plantar pressure measurements, the lack of assessor blinding was not considered a potential source of bias.

### Equipment

Plantar pressures beneath the foot were measured using the pedar®-X in-shoe plantar pressure system (Novel GmbH, Munich, Germany). The pedar®-X comprises of 99 capacitive sensors arranged in a grid and embedded within a thin flexible insole. The pedar®-X insoles were calibrated using the trublu® calibration device as per the manufacturer’s guidelines (Novel GmbH, Munich, Germany) prior to data collection. The sampling frequency of the system was 50 Hz. The pedar®-X is widely used in foot plantar pressure research [19–24] and has been demonstrated to be a valid and reliable in-shoe pressure measurement system [25–28]. It has high test-retest reliability with coefficients of repeatability for metatarsal head measurements of between 1.2 to 7.7% [27], and coefficients of variation for metatarsal head measurements of between 3.4 and 24.1% [28]. This equipment has also previously been used in similar projects with older people [12, 19, 29].

### Protocol

Participants were required to attend one test session that was of approximately 75 min duration. Screening for inclusion into the study was initially conducted by phone when the appointment was made for the test session. Eligibility was based on self-reporting by the participant of conditions from the inclusion and exclusion criteria. In the case of walking household distances, participants were asked this question over the phone and this was confirmed at their test session. If they were unable to walk from the waiting room to the research room where testing was conducted (a distance of 10 m) without a walking aid, they were excluded.

After confirmation that the participant had read and understood the participant information statement, all participants signed the informed consent form prior to commencing the data collection session. Once informed consent was obtained, standard demographic and participant information were collected on a data collection form. Following this, participants’ shoe size was determined and they were fitted with a pair of standardised, extra-depth shoes (Gadean Footwear, O’Connor, WA, Australia). The standardised shoe was used during all testing to control for influences of footwear on plantar pressures. The shoe had a sole hardness of Shore A 40 durometer, with a heel height of 27 mm and a forefoot height of 13 mm.

Cardboard insoles that were matched to the size of each participant’s shoes were then inserted into the shoe. The metatarsal heads and styloid process were palpated and marked with an ink pen. Next, the participant was asked to don the shoes, with the cardboard template inside. Following this, they were asked to stand up, allowing the ink to be transferred from the foot to the cardboard insole. This marked the position of each metatarsal head and the styloid process on the insole. The participant was then asked to take the shoes off so the cardboard template could be removed, thus allowing the outline of the metatarsal parabola to be marked on the insole. The three positions of the metatarsal pads (proximal, in-line, and distal) were measured on the template based on the metatarsal parabola (Table 1). The cardboard template (with or without a metatarsal dome) was then inserted back into the shoe.

Participants then had the pedar®-X equipment attached to them and connected ready for use. The appropriately sized pedar®-X insole was inserted into the shoe on top of the cardboard template, and the participant put the shoes on again, so the pedar®-X insole was positioned between the participant’s foot and the cardboard template. Participants were given sufficient time to acclimatise and be comfortable walking in the standardised shoes and with the pedar®-X equipment in place. Next, the participant was instructed to walk at their normal comfortable speed while being timed. To minimise the confounding effect of different walking speeds on the pressure data, a trial was repeated if the walking speed for each condition differed by more than 5% of the original walking speed. Four walking trials along an 8 m walkway were recorded for each test condition, with the middle four steps for each trial included in the analysis (to exclude acceleration and deceleration steps). The 16 steps (4 trials × 4 steps) were subsequently averaged for each test condition.

### Outcome measures

The primary outcome measure was peak pressure under the forefoot. The secondary outcome measures were maximum force at the time of peak pressure and contact area at the time of peak pressure under the forefoot. To cross-check walking speed, total contact time for each intervention was also measured.

### Data analysis

**Fig. 2:**
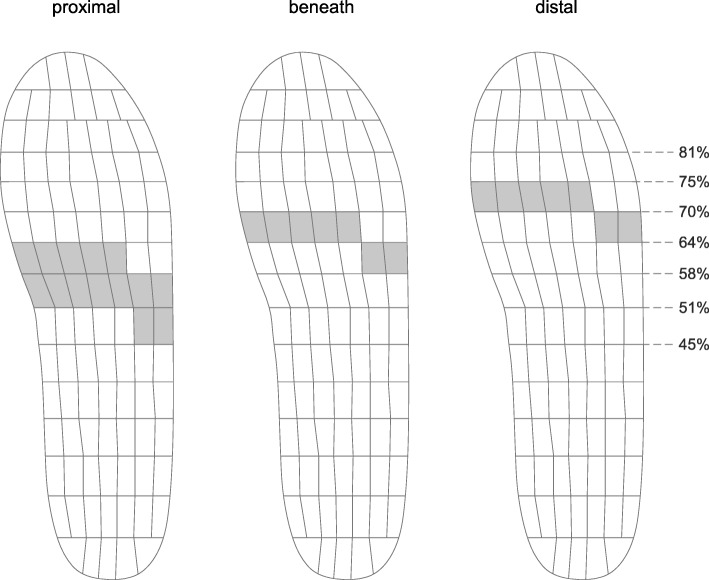
Anatomically-based masking protocol used in the study (from Forghany et al. [30])

The plantar pressure data were entered into the pedar® analysis program and a new, previously published anatomically-based masking protocol was used where there are three mask sub-areas of the forefoot according to their position relative to the metatarsal heads [30]. The three sub-areas were: (i) proximal to the metatarsal heads, (ii) beneath the metatarsal heads, and (iii) distal to the metatarsal heads (Fig. 2). This protocol has previously been published and was found to have excellent intra- and inter-rater reliability [30]. The reason for utilising this protocol was to enhance our analysis by being able to more precisely determine where force and contact area were being altered to achieve the plantar pressure reductions observed with the metatarsal domes. The same masking protocol was applied for each participant and for each trial to ensure consistency.

Data were analysed using IBM Statistical Package for the Social Sciences (SPSS) Version 25.0 (IBM Corporation, Armonk, NY). All data were explored for normality prior to inferential analysis. A one-way repeated-measures analysis of variance (ANOVA) with Bonferroni-adjusted post-hoc tests were used to compare means between each of the conditions. Differences between conditions were considered significant if *p* < 0.05. Where the data violated the assumption of sphericity in ANOVA (if *p* < 0.05 for Mauchley’s Test of Sphericity), the Greenhouse–Geisser correction was used to obtain the degrees of freedom and *p*-values for the *F*-statistic.

## Results

### Participants

Of the 36 participants recruited into the study, 31 were female (86%) and 5 were male (14%). The mean (SD) age was 75.5 (5.5) years, with a range of 65.1–88.5. Participant characteristics are shown in Table 2.

**Table 2:** Participant characteristics (*N* = 36)

Characteristic	Number (%), unless otherwise stated
Age in years – mean (SD), range	75.5 (5.5), 65.1–88.5
Sex – females	31 (86%)
Height – mean (SD)	1.62 (0.09)
Body weight – mean (SD)	74.3 (13.4)
BMI in kg/m^2^ – mean (SD)	28.4 (4.1)

### Walking speed

Because changes in walking speed can affect plantar pressures, we initially assessed whether walking speed changed between the experimental conditions by analysing for differences in contact time prior to statistical analysis. There was no significant difference for contact time between the 7 conditions (F_4.5, 157.4_ = 1.892, *p* = 0.106) indicating that the participants walked at a consistent speed for all conditions (Table 3). Therefore, any plantar pressure differences that were found can be directly attributed to the test condition, not due to changes in contact time.

**Table 3:** Contact time (*N* = 36)

Condition	Mean (ms)	SD (ms)
Control (no pad)	702.9	89.4
Emsold metatarsal dome proximal	700.7	90.6
Emsold metatarsal dome in-line	696.0	82.1
Emsold metatarsal dome distal	706.5	92.5
Langer PPT metatarsal pad proximal	696.0	84.3
Langer PPT metatarsal pad in-line	701.1	82.6
Langer PPT metatarsal pad distal	700.8	91.1

### Peak pressure

**Fig. 3:**
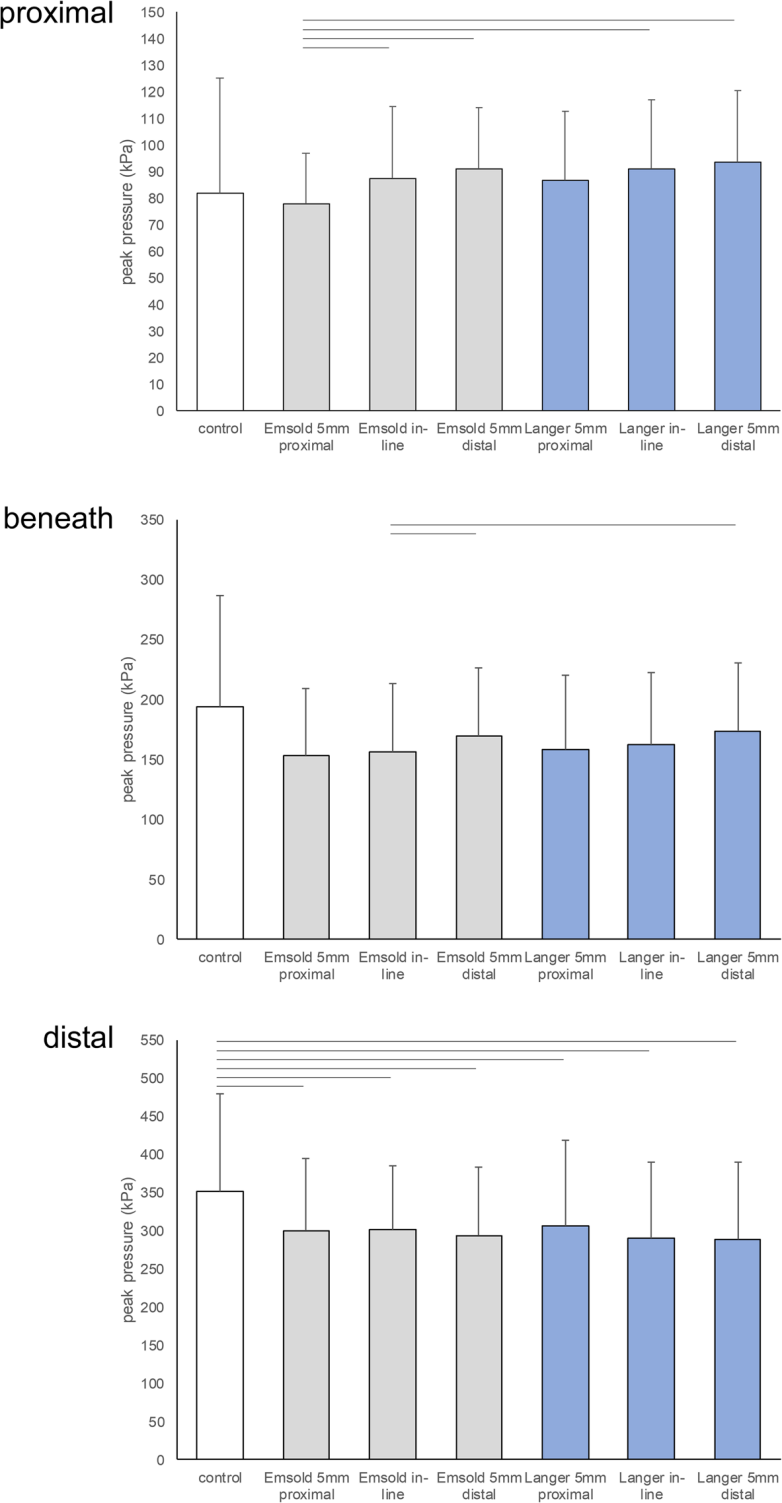
Graphic presentation of mean peak pressure (SD) in kilopascals (kPa) for each of the 7 conditions for the proximal, beneath and distal masks (bars at the top of each graph represent conditions that were significantly different, *p* < 0.05)

When analysed with the mask that was *proximal* to the metatarsal heads, where the bulk of the metatarsal dome was positioned, there was a significant effect for peak pressure at the forefoot between the 7 conditions (F_2.1, 73.5_ = 3.140, *p* = 0.047). While none of the metatarsal dome conditions significantly altered peak pressure when compared to the control (no pad) condition (Table 4 and Fig. 3), there were several significant differences between the metatarsal dome conditions (Additional file 1 presents pairwise comparisons) dependent on the position of the metatarsal dome (proximal, in-line or distal). Generally, the more proximal a metatarsal dome was positioned, the lower the plantar pressure was. The proximally positioned Emsold metatarsal dome provided the lowest plantar pressure compared to the in-line and distally positioned metatarsal domes (both Emsold and Langer). However, there was no significant difference in peak pressure between the proximally positioned Emsold metatarsal dome and the proximally positioned Langer PPT metatarsal pad.

**Table 4:** Mean peak pressure (SD) and comparisons in kilopascals (kPa) for each of the 7 conditions (*N* = 36)

Condition	Proximal mask			Beneath mask			Distal mask		
	Mean (SD)	Mean diff. (95% CI)	*P*-value	Mean (SD)	Mean diff. (95% CI)	*P*-value	Mean (SD)	Mean diff. (95% CI)	*P*-value
**1. Control**	81.7 (43.4)	N/A	N/A	193.7 (92.6)	N/A	N/A	351.0 (128.6)	N/A	N/A
**2. Emsold metatarsal dome 5 mm proximal**	77.6 (19.2) ^3, 4, 6, 7^	4.0 (−16.5, 24.5)	1.000	152.8 (56.4)	40.8 (−2.5, 84.2)	0.083	300.0 (94.6) ^1^	51.0 (1.8, 100.2)	0.036
**3. Emsold metatarsal dome in-line**	87.4 (26.9) ^2^	−5.7 (−24.1, 12.7)	1.000	156.1 (57.0) ^4, 7^	37.6 (−1.3, 76.5)	0.067	301.6 (83.7) ^1^	49.4 (7.9, 91.0)	0.009
**4. Emsold metatarsal dome 5 mm distal**	91.0 (23.2) ^2^	−9.4 (−27.8, 9.1)	1.000	169.9 (56.9) ^3^	23.8 (− 9.9, 57.4)	0.565	293.4 (90.4) ^1^	57.6 (11.4, 103.8)	0.005
**5. Langer PPT metatarsal pad 5 mm proximal**	86.6 (26.1)	−4.9 (−24.6, 14.7)	1.000	158.5 (62.1)	35.2 (−0.2, 70.6)	0.053	306.3 (112.5) ^1^	44.7 (7.4, 82.0)	0.008
**6. Langer PPT metatarsal pad in-line**	91.0 (25.9) ^2^	−9.3 (− 34.1, 15.5)	1.000	161.9 (60.8)	31.7 (−8.8, 72.3)	0.309	290.6 (99.5) ^1^	60.5 (19.9, 101.0)	< 0.001
**7. Langer PPT metatarsal pad 5 mm distal**	93.5 (26.7) ^2^	−11.8 (− 38.3, 14.7)	1.000	173.4 (57.6) ^3^	20.3 (− 21.2, 61.7)	1.000	289.1 (99.8) ^1^	61.9 (18.8, 105.1)	0.001

Notes: Proximal mask positioned proximal to the metatarsal heads; Beneath mask positioned beneath the metatarsal heads; Distal mask positioned distal to the metatarsal heads. Full results and pairwise comparisons for all conditions are contained in Additional file 1

Abbreviations: ^1^significantly different from control condition; ^2^ significantly different from Emsold proximal; ^3^ significantly different from Emsold in-line, ^4^ significantly different from Emsold distal; ^5^ significantly different from Langer proximal; ^6^ significantly different from Langer in-line, ^7^ significantly different from Langer distal

When analysed with the mask that was *beneath* the metatarsal heads, there was a significant effect for peak pressure at the forefoot between the 7 conditions (F_2.0, 70.4_ = 6.713, *p* = 0.002). While none of the metatarsal dome conditions significantly altered plantar pressure when compared to the control condition (Table 4 and Fig. 3), there were two significant differences between the metatarsal dome conditions (Additional file 1 presents pairwise comparisons). The in-line positioned Emsold metatarsal dome reduced plantar pressure more than the distally positioned Emsold metatarsal dome and the distally positioned Langer PPT metatarsal pad.

When analysed with the mask that was *distal* the metatarsal heads, where the highest plantar pressures were recorded, there was a significant effect for peak pressure at the forefoot between the 7 conditions (F_3.9, 135.6_ = 8.125, *p* < 0.001). All of the metatarsal dome conditions significantly reduced pressure when compared to the control condition (Table 4 and Fig. 3), but there were no significant differences between any of the metatarsal dome conditions (Additional file 1 presents pairwise comparisons). This reduction in plantar pressure was in the order of 45–60 kPa.

Summarising the peak pressure findings, the metatarsal domes led to significant reductions in plantar pressure at the forefoot. This reduction in plantar pressure was observed most in the mask that was distal to the metatarsal heads, where the plantar pressure readings were at their highest. For some of the metatarsal dome conditions this did not occur at the expense of increasing plantar pressure in the mask that was proximal to the metatarsal heads, where the bulk of the metatarsal dome was positioned; that is, peak plantar pressure was not simply moved from one area to another. Overall, there were no significant differences between the two metatarsal domes (i.e. Emsold metatarsal dome and Langer PPT metatarsal pad) in their ability to reduce plantar pressure when both were positioned proximally. However, the proximally positioned Emsold metatarsal dome was found to lead to the best combination plantar pressure redistribution.

### Maximum force

**Fig. 4:**
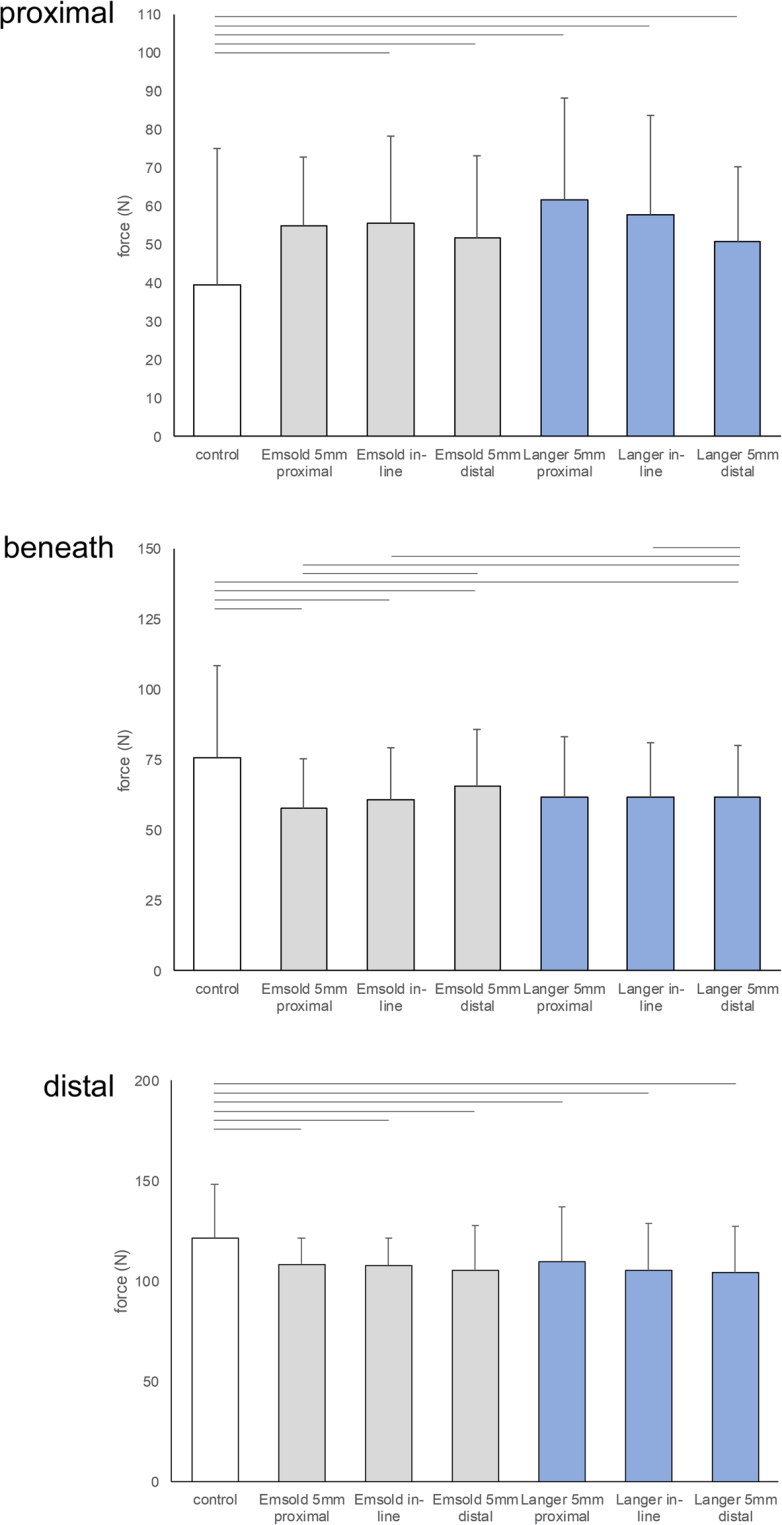
Graphic presentation of mean force (SD) in Newtons (N) at the time of peak pressure for each of the 7 conditions for the proximal, beneath and distal masks (bars at the top of each graph represent conditions that were significantly different, *p* < 0.05)

When analysed with the mask that was *proximal* to the metatarsal heads, where the bulk of the metatarsal dome was positioned, there was a significant effect for maximum force at the forefoot between the 7 conditions (F_2.2, 78.4_ = 6.332, *p* = 0.002). Three of the metatarsal dome conditions significantly increased maximum force when compared to the control condition (Table 5 and Fig. 4); the in-line positioned Emsold metatarsal dome, the distally positioned Emsold metatarsal dome, and the proximally positioned Langer PPT metatarsal pad. There were no significant differences between any of the pad conditions (Additional file 2 presents pairwise comparisons).

**Table 5:** Mean maximum force (SD) and comparisons in Newtons (N) at the time of peak pressure for each of the 7 conditions (*N* = 36)

Condition	Proximal mask			Beneath mask			Distal mask		
	Mean (SD)	Mean diff. (95% CI)	*P*-value	Mean (SD)	Mean diff. (95% CI)	*P*-value	Mean (SD)	Mean diff. (95% CI)	*P*-value
**1. Control**	39.5 (35.4)	N/A	N/A	75.5 (33.0)	N/A	N/A	121.3 (26.8)	N/A	N/A
**2. Emsold metatarsal dome 5 mm proximal**	54.9 (17.8)	−15.4 (−32.9, 2.1)	0.141	57.9 (17.5) ^1, 4, 7^	17.6 (2.9, 32.3)	0.008	108.3 (23.3) ^1^	13.0 (3.0, 23.1)	0.003
**3. Emsold metatarsal dome in-line**	55.6 (22.6) ^1^	−16.1 (−31.1, − 1.2)	0.025	60.7 (18.6) ^1, 7^	14.8 (2.2, 27.5)	0.011	107.6 (23.1) ^1^	13.7 (6.3, 21.1)	< 0.001
**4. Emsold metatarsal dome 5 mm distal**	51.5 (21.5) ^1^	−11.9 (−22.9, −0.9)	0.024	65.7 (20.0) ^2^	9.9 (−0.8, 20.5)	0.095	105.1 (22.4) ^1^	16.2 (8.0, 24.3)	< 0.001
**5. Langer PPT metatarsal pad 5 mm proximal**	61.6 (26.5) ^1^	−22.1 (−36.6, −7.5)	< 0.001	61.7 (21.6) ^1^	13.8 (2.6, 25.0)	0.006	109.6 (27.4) ^1^	11.7 (4.4, 18.9)	< 0.001
**6. Langer PPT metatarsal pad in-line**	57.8 (25.7)	−18.3 (− 39.3, 2.8)	0.155	61.8 (18.9) ^7^	13.7 (−0.3, 27.8)	0.061	105.1 (23.7) ^1^	16.2 (8.7, 23.7)	< 0.001
**7. Langer PPT metatarsal pad 5 mm distal**	50.6 (19.7)	−11.1 (−31.3, 9.1)	1.000	67.1 (18.2) ^2, 3, 6^	8.4 (−5.9, 22.8)	1.000	104.3 (22.8) ^1^	17.0 (9.0, 25.1)	< 0.001

Notes: Proximal mask positioned proximal to the metatarsal heads; Beneath mask positioned beneath the metatarsal heads; Distal mask positioned distal to the metatarsal heads. Full results and pairwise comparisons for all conditions are contained in Additional file 2

Abbreviations: ^1^ significantly different from control condition; ^2^ significantly different from Emsold proximal; ^3^ significantly different from Emsold in-line, ^4^ significantly different from Emsold distal; ^5^ significantly different from Langer proximal; ^6^ significantly different from Langer in-line, ^7^ significantly different from Langer distal

When analysed with the mask that was *beneath* the metatarsal heads mask, there was a significant effect for maximum force at the forefoot between the 7 conditions (F_2.3, 81.4_ = 9.049, *p* < 0.001). Three of the metatarsal dome conditions significantly decreased maximum force when compared to the control condition (Table 5 and Fig. 4); the proximally positioned Emsold metatarsal dome, the in-line positioned Emsold metatarsal dome, and the proximally positioned Langer PPT metatarsal pad. There were several significant differences between the metatarsal dome conditions (Additional file 2 presents pairwise comparisons) dependent on the type of metatarsal dome (Emsold or Langer) and the position of the metatarsal dome (proximal, in-line or distal). The proximally positioned Emsold metatarsal dome led to a significantly larger decrease in maximum force when compared to the distally positioned Emsold metatarsal dome and the distally positioned Langer PPT metatarsal pad. In addition, the in-line positioned Emsold metatarsal dome and the in-line positioned Langer PPT led to a significantly larger decrease in maximum force when compared to the distally positioned Langer PPT metatarsal pad.

When analysed with the mask that was *distal* to the metatarsal heads mask, where the highest forces were recorded, there was a significant effect for maximum force at the forefoot between the 7 conditions (F_4.3, 149.4_ = 13.365, *p* < 0.001). All of the metatarsal dome conditions significantly reduced maximum force when compared to the control condition (Table 5 and Fig. 4), but there were no significant differences between any of the metatarsal dome conditions (Additional file 2 presents pairwise comparisons). This reduction in force was in the order of 12–17 N.

Summarising the maximum force findings, the metatarsal domes significantly reduced maximum force in the mask that was distal to the metatarsal heads, where the force readings were at their highest. For some of the metatarsal dome conditions, this did not occur at the expense of significantly increasing force in the mask that was proximal to the metatarsal heads, where the bulk of the metatarsal dome was positioned; that is, maximum force was not simply moved from one area to another. Overall, the proximally positioned Emsold metatarsal dome was found to lead to the best combination of significantly decreasing maximum force distal to the metatarsal heads, but not adversely increasing maximum force proximally.

### Contact area

**Fig. 5:**
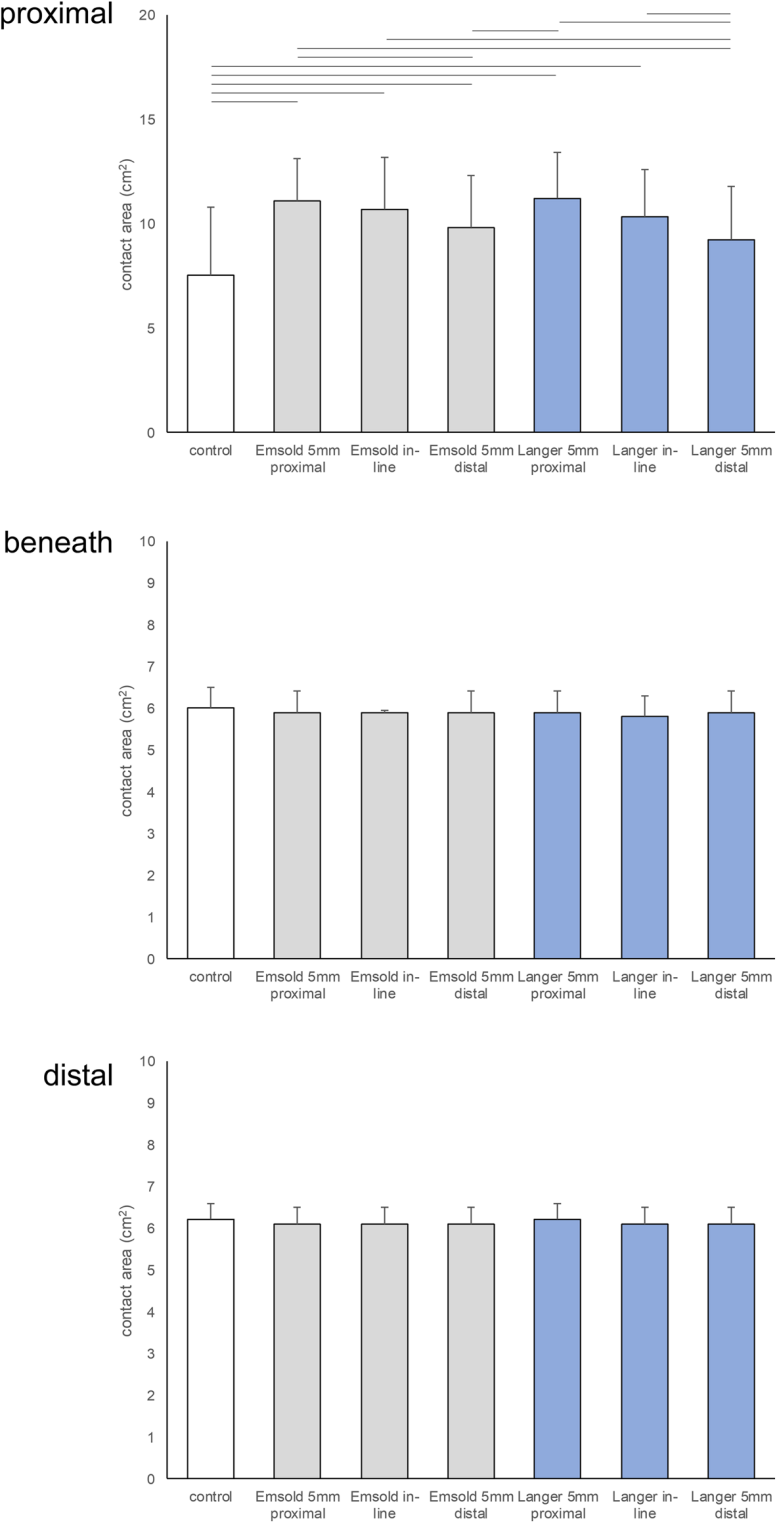
Graphic presentation of mean contact area (SD) in cm^2^ at the time of peak pressure for each of the 7 conditions for the proximal, beneath and distal masks (bars at the top of each graph represent conditions that were significantly different, *p* < 0.05)

When analysed with the mask that was *proximal* to the metatarsal heads, where the bulk of the metatarsal dome was positioned, there was a significant effect for contact area at the forefoot between the 7 conditions (F_3.3, 115.5_ = 23.600, *p* < 0.001) (Table 6 and Fig. 5). There were several significant differences between the metatarsal dome conditions (Additional file 3 presents pairwise comparisons) dependent on the type of metatarsal dome (Emsold or Langer) and the position of the metatarsal dome (proximal, in-line or distal). For both the Emsold metatarsal dome and the Langer PPT metatarsal pad, the more proximal the metatarsal dome was positioned, the greater the contact area at the forefoot. The proximally positioned Emsold metatarsal dome led to significantly greater increase in contact area compared to the distally positioned Emsold metatarsal dome and the distally positioned Langer PPT metatarsal pad. The in-line positioned Emsold metatarsal dome led to significantly greater increase in contact area compared to the distally positioned Langer PPT metatarsal pad. The proximally positioned Langer PPT metatarsal pad led to significantly greater increase in contact area compared to the distally positioned Emsold metatarsal dome and the distally positioned Langer PPT metatarsal pad. The in-line positioned Langer PPT metatarsal pad led to significantly greater increase in contact area compared to the distally positioned Langer PPT metatarsal pad. These increases in contact area relative were in the order of 2–4 cm^2^.

**Table 6:** Mean contact area (SD) and comparisons in cm^2^ at the time of peak pressure for each of the 7 conditions (*N* = 36)

Condition	Proximal mask			Beneath mask			Distal mask		
	Mean (SD)	Mean diff. (95% CI)	*P*-value	Mean (SD)	Mean diff. (95% CI)	*P*-value	Mean (SD)	Mean diff. (95% CI)	*P*-value
**1. Control**	7.5 (3.3)	N/A	N/A	6.0 (0.5)	N/A	N/A	6.2 (0.4)	N/A	N/A
**2. Emsold metatarsal dome 5 mm proximal**	11.1 (2.0) ^1, 4, 7^	−3.6 (−5.0, − 2.1)	< 0.001	5.9 (0.5)	0.1 (− 0.1, 0.3)	1.000	6.1 (0.4)	0.1 (− 0.1, 0.2)	1.000
**3. Emsold metatarsal dome in-line**	10.7 (2.5) ^1, 7^	−3.1 (−4.5, − 1.8)	< 0.001	5.9 (0.4)	0.1 (− 0.1, 0.3)	1.000	6.1 (0.4)	0.1 (−0.1, 0.2)	1.000
**4. Emsold metatarsal dome 5 mm distal**	9.8 (2.5) ^1, 2, 5^	−2.2 (−3.4, −1.1)	< 0.001	5.9 (0.5)	0.1 (− 0.1, 0.3)	1.000	6.1 (0.4)	0.0 (−0.1, 0.1)	1.000
**5. Langer PPT metatarsal pad 5 mm proximal**	11.2 (2.2) ^1, 4, 7^	−3.7 (−4.9, −2.5)	< 0.001	5.9 (0.5)	0.1 (−0.1, 0.3)	1.000	6.2 (0.4)	0.0 (0.0, 0.0)	1.000
**6. Langer PPT metatarsal pad in-line**	10.3 (2.3) ^1, 7^	−2.8 (−4.6, −1.0)	< 0.001	5.8 (0.5)	0.2 (−0.1, 0.4)	0.293	6.1 (0.4)	0.0 (0.0, 0.1)	1.000
**7. Langer PPT metatarsal pad 5 mm distal**	9.2 (2.6) ^2, 3, 5, 6^	−1.6 (−3.4, 0.1)	0.081	5.9 (0.5)	0.1 (−0.1, 0.2)	1.000	6.1 (0.4)	0.1 (−0.1, 0.2)	1.000

Notes: Proximal mask positioned proximal to the metatarsal heads; Beneath mask positioned beneath the metatarsal heads; Distal mask positioned distal to the metatarsal heads. Full results and pairwise comparisons for all conditions are contained in Additional file 3 (contact area results are presented to 3 decimal places in Additional file 3)

Abbreviations: ^1^ significantly different from control condition; ^2^ significantly different from Emsold proximal; ^3^ significantly different from Emsold in-line, ^4^ significantly different from Emsold distal; ^5^ significantly different from Langer proximal; ^6^ significantly different from Langer in-line, ^7^ significantly different from Langer distal

There were no significant effects for contact area at the forefoot between the 7 conditions when analysed with the mask that was *beneath* to the metatarsal heads (F_4.6, 159.6_ = 1.840, *p* = 0.115) and with the mask that was *distal* to the metatarsal heads (F_6.0, 210.0_ = 1.448, *p =* 0.198). These results are presented in Table 6 and Fig. 5 (Additional file 3 presents pairwise comparisons).

Summarising the contact area findings, the metatarsal domes significantly increased contact area in the mask that was proximal to the metatarsal heads, where the bulk of the metatarsal dome was positioned. The proximally positioned Emsold metatarsal dome and the proximally positioned Langer PPT metatarsal pad led to the largest increases in contact area at the forefoot.

## Discussion

The aim of this study was to evaluate the effect on plantar pressure of metatarsal domes (the *Emsold metatarsal dome* and the *Langer PPT metatarsal pad*) in different positions in older people with a history of forefoot pain. To achieve this, and to help explain how the metatarsal domes produce the effects observed, we first developed a new anatomically-based masking protocol [30], which separated the forefoot into three distinct mask regions (proximal to the metatarsal heads, beneath the metatarsal heads, and distal to the metatarsal heads). We chose to develop this method because in a previous study that we conducted [12], which evaluated the effects of different forefoot pads on plantar pressure, we could not accurately determine where in the forefoot peak pressure was reduced, nor could we adequately explain how the metatarsal domes achieved their effect.

The results of our current study show that the metatarsal domes did reduce forefoot plantar pressure in comparison to the control (no metatarsal dome) condition, and this was most notable – in the order of 45–60 kPa – under the area of the forefoot where the highest plantar pressures were recorded, which was distal to the metatarsal heads. This reduction in plantar pressure should be beneficial where high plantar pressures are associated with forefoot pain. However, this beneficial offloading may be negated if at the same time the metatarsal dome adversely increases plantar pressure more proximally where the bulk of the metatarsal dome is positioned. With this in mind, the best combination of offloading is where a metatarsal dome significantly reduces plantar pressure distally, where the highest plantar pressures are, but does not significantly increase plantar pressures proximally, where the bulk of the metatarsal dome is positioned. Our data demonstrates that two of the metatarsal dome conditions achieved this – the Emsold metatarsal dome positioned proximally and the Langer PPT metatarsal pad positioned proximally. Although, when our data for the proximally positioned metatarsal domes is more closely evaluated, the Emsold metatarsal dome was more effective than the Langer PPT metatarsal pad.

This superior performance of the Emsold metatarsal dome compared to the Langer PPT metatarsal pad can be explained by the hardness of the two metatarsal domes. The Emsold metatarsal dome (Shore A hardness of 11 durometer) is relatively ‘softer’ than the Langer PPT metatarsal pad (Shore A hardness of 20 durometer). This relative softness leads to a better combination of plantar pressure reduction distally but not causing an adverse increase in plantar pressure proximally. Put simply, the softer Emsold metatarsal dome may subtly mould more to the foot in the region proximal to the metatarsal heads, thus causing no increase in plantar pressure at this site, while still decreasing plantar pressure where it is most needed, which is distal to the metatarsal heads where the highest plantar pressures are found. It is plausible that this characteristic of the Emsold metatarsal dome would be important for comfort and wearability, although further studies are needed to determine this for certain.

Interestingly, while all the metatarsal dome conditions that we tested were found to decrease plantar pressure distally, four significantly increased plantar pressure proximally, which as inferred previously, may lead to comfort issues when using the metatarsal domes in these positions (i.e. potential irritation of the skin adjacent to where the pad is positioned). These conditions included the Emsold metatarsal dome and Langer PPT metatarsal pad when positioned in-line or distal to the metatarsal heads. These findings indicate that metatarsal domes should not be positioned in-line or distal to the metatarsal heads; instead, the best position for them is where their anterior border is proximal to the metatarsal parabola (i.e. the line representing the metatarsal heads). In our study this position was 5 mm proximal to the metatarsal parabola, so until further studies are done that indicate otherwise, we recommend this position as being the most effective position.

The anatomically-based masking protocol that we used for this study [30] provided data to enhance our understanding of the mechanism of action of the metatarsal dome. To achieve a reduction in plantar pressure, forefoot pads must reduce force and/or increase contact area on the plantar surface of the foot [20, 21]. We found that the metatarsal domes tested in our current study, particularly when positioned proximal to the metatarsal heads, reduced plantar pressure in the forefoot by both decreasing force and increasing contact area. They achieve this by the shape and location of the pad, which reduces force distal to the second, third and fourth metatarsal heads by redistributing some of that force proximally. The pad achieves this by increasing the area over which the force is distributed. Because pressure is equal to force divided by area, any decrease in force or increase in contact area by the metatarsal dome will lead to a decrease in plantar pressure at the forefoot. While this finding is intuitive, this study is the first to demonstrate this in a systematic way by using the anatomically-based masking protocol.

The finding that more proximally positioned forefoot pads reduce plantar pressure more than distally positioned forefoot pads contrasts with our earlier study [12]. In that study, we found that a Langer PPT metatarsal pad positioned 5 mm distal to the metatarsal heads reduced plantar pressure more in the forefoot than a similar pad positioned 10 mm proximal to the metatarsal heads. This finding was most likely due to the plantar pressure masking technique used in that study, which was a single, relatively large mask that represented the entire forefoot, rather than the three smaller anatomically-based masks that we used in the current study (however, also note that in the current study we positioned this pad 5 mm proximal to the metatarsal heads, not 10 mm proximal). The anatomically-based masking protocol, therefore, enables a better explanation, in a dose-response manner, of the changes in plantar pressure (i.e. force and contact area) between various positions of metatarsal domes. Put simply, the simple forefoot mask protocol that we used in our earlier study did not provide us with the ability to distinguish more subtle plantar pressure changes with the forefoot pads compared to the anatomically-based masking protocol that we used in our current study.

Reductions in plantar pressure measured in our study were in the order of 45–60 kPa or approximately 13 to 17% (distal mask analysis), which is similar to what we found in our earlier study [12]. Similar reductions have also been found in other in-shoe plantar pressure studies of forefoot padding [10, 13, 15, 22, 31–33], although some of the pads in these studies are somewhat different to the pads we used and were tested on different populations (e.g. participants with diabetes or rheumatoid arthritis). This highlights that our new anatomically-based masking protocol is capable of detecting plantar pressure changes of similar magnitude to other protocols, but it has the added benefit of being able to explain how these plantar pressure changes are produced.

It has been shown that redistributing plantar pressure in the forefoot can lead to a reduction in forefoot pain [4]. However, it is not known whether a difference of approximately 13 to 17% – the reductions found in this study – is sufficient to reduce pressure-related forefoot pain. One study [11] reported that pain relief occurred when plantar pressure was reduced by approximately 12%, however this study was performed on a younger population (mean age approximately 50 years compared to the mean age of our sample of approximately 75 years). Randomised trials where both plantar pressure and pain are measure simultaneously are needed to ascertain this.

This study has four strengths. Firstly, previous studies have investigated the effects of different positions of metatarsal pads [13–15], however none of these studies have specifically been conducted on older people with forefoot pain. Secondly, this study compared commercially available prefabricated metatarsal domes of different densities (i.e. hardnesses), and as such, our findings reflect clinical practice. However, our study is difficult to compare to others as they used metatarsal domes of different densities to the ones used in our study, and some studies used pads made from other materials such as cork, felt or foam [13–15]. Thirdly, we elected to perform data analysis using a new anatomically-based masking protocol for the forefoot region (rather than mask the entire forefoot), which provided us with the ability to explain more effectively the mechanism of action of the forefoot pads. Further, our new masking technique has an advantage over attempting to mask individual metatarsal heads because of the poor reliability associated of that technique [34] and because older people having a high level of forefoot deformities making determination of the position of individual metatarsal heads difficult [1, 35, 36]. Finally, the methods of our study were pragmatic as the protocol for the placement of forefoot pads was designed to reflect clinical practice.

There are, however, two limitations to this study that should be taken into account. Firstly, this study only evaluated plantar pressure, and although increased plantar pressure has been associated with forefoot pain in older people, randomised trials using patient-reported outcome measures are needed to evaluate whether forefoot pads do indeed reduce forefoot pain in older people, and to determine whether they are comfortable and wearable. Secondly, although the pedar®-X has been shown to be a valid and reliable plantar pressure system it only records forces that are applied perpendicular to the pressure sensors [25–28]. Accordingly, the shear component of forces acting at the interface between the metatarsal pad and the sensor is unable to be determined [37, 38]. Furthermore, the contact surface of the forefoot pads that were used in this study are curvilinear but the sensors are calibrated when placed flat. As a result, it is possible that inherent measurement error occurs, but the magnitude of any such error is currently unknown. Potential accuracy errors have been shown to exist with plantar measuring systems when measuring contact area, otherwise referred to as spatial resolution [39–41]. Despite these limitations, in-shoe pressure measuring systems are considered the best available method for measuring forces acting between in-shoe devices and the foot [37, 38] and they have been in common use in the last two decades to evaluate the mechanical effects of in-shoe devices and shoes in older populations [12, 19, 29, 42], as well as in people with degenerative disorders [43, 44], rheumatoid arthritis [23, 31] and diabetic peripheral neuropathy [45, 46].

## Conclusion

The results of this study indicate that metatarsal domes reduce plantar pressure in the forefoot in older people with a history of forefoot pain. All metatarsal dome conditions significantly reduced peak pressure in the forefoot, however metatarsal domes that were positioned 5 mm proximal to the metatarsal heads provided the best balance of reducing plantar pressure distal to the metatarsal heads, where the pressure is at its greatest, but not adversely increasing pressure proximally where the bulk of the pad is positioned. When positioned 5 mm proximal to the metatarsal heads, the Emsold metatarsal dome was more effective than the Langer PPT metatarsal pad, so we cautiously recommend this forefoot pad for alleviating plantar pressure in older people with a history of forefoot pain.

## Supplementary information

**Additional file 1. **Pairwise comparisons for mean peak pressure (kPa).

**Additional file 2. **Pairwise comparisons for mean force (N) at time of peak pressure.

**Additional file 3. **Pairwise comparisons for contact area (cm^2^) at time of peak pressure.

## Data Availability

The datasets used and/or analysed during the current study are available from the corresponding author upon reasonable request.
