# Supplementary material for: Effects of metatarsal domes on plantar pressures in older people with a history of forefoot pain
Source: J Foot Ankle Res. 2020 May 6;13:18. doi: 10.1186/s13047-020-00388-x (PMC7201604; doi:10.1186/s13047-020-00388-x)
Supplement: Supplementary file 1 — Additional file 1. Pairwise comparisons for mean peak pressure (kPa). [file 13047_2020_388_MOESM1_ESM.docx]

**Additional file 1A** Pairwise comparisons for mean peak pressure (kPA) for the proximal mask (N = 36)

| **Condition** | **Comparison** | **Mean difference^*^** | **95% CI^†^** | | ***P*-value^†^** |
| --- | --- | --- | --- | --- | --- |
|  | **condition** |  | **Lower limit** | **Upper limit** |  |
| **1** | 2 | 4.028 | -16.463 | 24.519 | 1.000 |
|  | 3 | -5.694 | -24.098 | 12.709 | 1.000 |
|  | 4 | -9.375 | -27.842 | 9.092 | 1.000 |
|  | 5 | -4.931 | -24.563 | 14.701 | 1.000 |
|  | 6 | -9.306 | -34.086 | 15.475 | 1.000 |
|  | 7 | -11.806 | -38.293 | 14.682 | 1.000 |
| **2** | 1 | -4.028 | -24.519 | 16.463 | 1.000 |
|  | 3 | -9.722^*^ | -17.943 | -1.502 | 0.009 |
|  | 4 | -13.403^*^ | -22.055 | -4.751 | <0.001 |
|  | 5 | -8.958 | -19.840 | 1.924 | 0.225 |
|  | 6 | -13.333^*^ | -25.201 | -1.466 | 0.016 |
|  | 7 | -15.833^*^ | -28.184 | -3.482 | 0.004 |
| **3** | 1 | 5.694 | -12.709 | 24.098 | 1.000 |
|  | 2 | 9.722^*^ | 1.502 | 17.943 | 0.009 |
|  | 4 | -3.681 | -13.190 | 5.829 | 1.000 |
|  | 5 | 0.764 | -6.901 | 8.428 | 1.000 |
|  | 6 | -3.611 | -14.003 | 6.781 | 1.000 |
|  | 7 | -6.111 | -19.298 | 7.076 | 1.000 |
| **4** | 1 | 9.375 | -9.092 | 27.842 | 1.000 |
|  | 2 | 13.403^*^ | 4.751 | 22.055 | <0.001 |
|  | 3 | 3.681 | -5.829 | 13.190 | 1.000 |
|  | 5 | 4.444 | -7.663 | 16.552 | 1.000 |
|  | 6 | 0.069 | -13.748 | 13.887 | 1.000 |
|  | 7 | -2.431 | -14.636 | 9.775 | 1.000 |
| **5** | 1 | 4.931 | -14.701 | 24.563 | 1.000 |
|  | 2 | 8.958 | -1.924 | 19.840 | 0.225 |
|  | 3 | -0.764 | -8.428 | 6.901 | 1.000 |
|  | 4 | -4.444 | -16.552 | 7.663 | 1.000 |
|  | 6 | -4.375 | -12.721 | 3.971 | 1.000 |
|  | 7 | -6.875 | -18.389 | 4.639 | 1.000 |
| **6** | 1 | 9.306 | -15.475 | 34.086 | 1.000 |
|  | 2 | 13.333^*^ | 1.466 | 25.201 | 0.016 |
|  | 3 | 3.611 | -6.781 | 14.003 | 1.000 |
|  | 4 | -0.069 | -13.887 | 13.748 | 1.000 |
|  | 5 | 4.375 | -3.971 | 12.721 | 1.000 |
|  | 7 | -2.500 | -11.429 | 6.429 | 1.000 |
| **7** | 1 | 11.806 | -14.682 | 38.293 | 1.000 |
|  | 2 | 15.833^*^ | 3.482 | 28.184 | 0.004 |
|  | 3 | 6.111 | -7.076 | 19.298 | 1.000 |
|  | 4 | 2.431 | -9.775 | 14.636 | 1.000 |
|  | 5 | 6.875 | -4.639 | 18.389 | 1.000 |
|  | 6 | 2.500 | -6.429 | 11.429 | 1.000 |

Notes: Results are based on estimated marginal means. *Mean differences significant at the 0.05 level. ^†^Adjustment for multiple comparisons: Bonferroni.

Conditions: ^1^ Control condition; ^2^ Emsold metatarsal dome 5 mm proximal to metatarsal heads; ^3^ Emsold metatarsal dome in-line with metatarsal heads, ^4^ Emsold metatarsal dome 5 mm distal to metatarsal heads; ^5^ Langer metatarsal dome 5 mm proximal to metatarsal heads; ^6^ Langer metatarsal dome in-line with metatarsal heads, ^7^ Langer metatarsal dome 5 mm distal to metatarsal heads.

**Additional file 1B** Pairwise comparisons for mean peak pressure (kPA) for the beneath mask (N = 36)

| **Condition** | **Comparison** | **Mean difference^*^** | **95% CI**^†^ | | ***P*-value**^†^ |
| --- | --- | --- | --- | --- | --- |
|  | **condition** |  | **Lower limit** | **Upper limit** |  |
| **1** | 2 | 40.833 | -2.495 | 84.161 | 0.083 |
|  | 3 | 37.569 | -1.318 | 76.457 | 0.067 |
|  | 4 | 23.750 | -9.934 | 57.434 | 0.565 |
|  | 5 | 35.208 | -0.227 | 70.644 | 0.053 |
|  | 6 | 31.736 | -8.779 | 72.252 | 0.309 |
|  | 7 | 20.278 | -21.161 | 61.717 | 1.000 |
| **2** | 1 | -40.833 | -84.161 | 2.495 | 0.083 |
|  | 3 | -3.264 | -15.984 | 9.456 | 1.000 |
|  | 4 | -17.083 | -37.528 | 3.362 | 0.203 |
|  | 5 | -5.625 | -22.542 | 11.292 | 1.000 |
|  | 6 | -9.097 | -25.789 | 7.594 | 1.000 |
|  | 7 | -20.556 | -41.638 | 0.527 | 0.062 |
| **3** | 1 | -37.569 | -76.457 | 1.318 | 0.067 |
|  | 2 | 3.264 | -9.456 | 15.984 | 1.000 |
|  | 4 | -13.819^*^ | -27.595 | -0.044 | 0.049 |
|  | 5 | -2.361 | -15.322 | 10.600 | 1.000 |
|  | 6 | -5.833 | -19.337 | 7.671 | 1.000 |
|  | 7 | -17.292^*^ | -32.790 | -1.793 | 0.018 |
| **4** | 1 | -23.750 | -57.434 | 9.934 | 0.565 |
|  | 2 | 17.083 | -3.362 | 37.528 | 0.203 |
|  | 3 | 13.819^*^ | 0.044 | 27.595 | 0.049 |
|  | 5 | 11.458 | -7.011 | 29.928 | 1.000 |
|  | 6 | 7.986 | -11.641 | 27.614 | 1.000 |
|  | 7 | -3.472 | -18.798 | 11.853 | 1.000 |
| **5** | 1 | -35.208 | -70.644 | 0.227 | 0.053 |
|  | 2 | 5.625 | -11.292 | 22.542 | 1.000 |
|  | 3 | 2.361 | -10.600 | 15.322 | 1.000 |
|  | 4 | -11.458 | -29.928 | 7.011 | 1.000 |
|  | 6 | -3.472 | -15.228 | 8.284 | 1.000 |
|  | 7 | -14.931 | -33.238 | 3.377 | 0.239 |
| **6** | 1 | -31.736 | -72.252 | 8.779 | 0.309 |
|  | 2 | 9.097 | -7.594 | 25.789 | 1.000 |
|  | 3 | 5.833 | -7.671 | 19.337 | 1.000 |
|  | 4 | -7.986 | -27.614 | 11.641 | 1.000 |
|  | 5 | 3.472 | -8.284 | 15.228 | 1.000 |
|  | 7 | -11.458 | -26.794 | 3.878 | 0.410 |
| **7** | 1 | -20.278 | -61.717 | 21.161 | 1.000 |
|  | 2 | 20.556 | -0.527 | 41.638 | 0.062 |
|  | 3 | 17.292^*^ | 1.793 | 32.790 | 0.018 |
|  | 4 | 3.472 | -11.853 | 18.798 | 1.000 |
|  | 5 | 14.931 | -3.377 | 33.238 | 0.239 |
|  | 6 | 11.458 | -3.878 | 26.794 | 0.410 |

Notes: Results are based on estimated marginal means. *Mean differences significant at the 0.05 level. ^†^Adjustment for multiple comparisons: Bonferroni.

Conditions: ^1^ Control condition; ^2^ Emsold metatarsal dome 5 mm proximal to metatarsal heads; ^3^ Emsold metatarsal dome in-line with metatarsal heads, ^4^ Emsold metatarsal dome 5 mm distal to metatarsal heads; ^5^ Langer metatarsal dome 5 mm proximal to metatarsal heads; ^6^ Langer metatarsal dome in-line with metatarsal heads, ^7^ Langer metatarsal dome 5 mm distal to metatarsal heads.

**Additional file 1C** Pairwise comparisons for mean peak pressure (kPA) for the distal mask (N = 36)

| **Condition** | **Comparison** | **Mean difference^*^** | **95% CI**^†^ | | ***P*-value**^†^ |
| --- | --- | --- | --- | --- | --- |
|  | **condition** |  | **Lower limit** | **Upper limit** |  |
| **1** | 2 | 51.042^*^ | 1.838 | 100.246 | 0.036 |
|  | 3 | 49.444^*^ | 7.929 | 90.960 | 0.009 |
|  | 4 | 57.639^*^ | 11.438 | 103.840 | 0.005 |
|  | 5 | 44.722^*^ | 7.446 | 81.999 | 0.008 |
|  | 6 | 60.486^*^ | 19.947 | 101.025 | <0.001 |
|  | 7 | 61.944^*^ | 18.781 | 105.108 | 0.001 |
| **2** | 1 | -51.042^*^ | -100.246 | -1.838 | 0.036 |
|  | 3 | -1.597 | -36.498 | 33.304 | 1.000 |
|  | 4 | 6.597 | -26.678 | 39.873 | 1.000 |
|  | 5 | -6.319 | -35.403 | 22.764 | 1.000 |
|  | 6 | 9.444 | -17.278 | 36.167 | 1.000 |
|  | 7 | 10.903 | -26.952 | 48.758 | 1.000 |
| **3** | 1 | -49.444^*^ | -90.960 | -7.929 | 0.009 |
|  | 2 | 1.597 | -33.304 | 36.498 | 1.000 |
|  | 4 | 8.194 | -17.410 | 33.799 | 1.000 |
|  | 5 | -4.722 | -42.746 | 33.302 | 1.000 |
|  | 6 | 11.042 | -17.741 | 39.825 | 1.000 |
|  | 7 | 12.500 | -14.240 | 39.240 | 1.000 |
| **4** | 1 | -57.639^*^ | -103.840 | -11.438 | 0.005 |
|  | 2 | -6.597 | -39.873 | 26.678 | 1.000 |
|  | 3 | -8.194 | -33.799 | 17.410 | 1.000 |
|  | 5 | -12.917 | -42.837 | 17.004 | 1.000 |
|  | 6 | 2.847 | -26.689 | 32.384 | 1.000 |
|  | 7 | 4.306 | -22.514 | 31.125 | 1.000 |
| **5** | 1 | -44.722^*^ | -81.999 | -7.446 | 0.008 |
|  | 2 | 6.319 | -22.764 | 35.403 | 1.000 |
|  | 3 | 4.722 | -33.302 | 42.746 | 1.000 |
|  | 4 | 12.917 | -17.004 | 42.837 | 1.000 |
|  | 6 | 15.764 | -10.046 | 41.574 | 1.000 |
|  | 7 | 17.222 | -18.951 | 53.396 | 1.000 |
| **6** | 1 | -60.486^*^ | -101.025 | -19.947 | <0.001 |
|  | 2 | -9.444 | -36.167 | 17.278 | 1.000 |
|  | 3 | -11.042 | -39.825 | 17.741 | 1.000 |
|  | 4 | -2.847 | -32.384 | 26.689 | 1.000 |
|  | 5 | -15.764 | -41.574 | 10.046 | 1.000 |
|  | 7 | 1.458 | -27.692 | 30.609 | 1.000 |
| **7** | 1 | -61.944^*^ | -105.108 | -18.781 | 0.001 |
|  | 2 | -10.903 | -48.758 | 26.952 | 1.000 |
|  | 3 | -12.500 | -39.240 | 14.240 | 1.000 |
|  | 4 | -4.306 | -31.125 | 22.514 | 1.000 |
|  | 5 | -17.222 | -53.396 | 18.951 | 1.000 |
|  | 6 | -1.458 | -30.609 | 27.692 | 1.000 |

Notes: Results are based on estimated marginal means. *Mean differences significant at the 0.05 level. ^†^Adjustment for multiple comparisons: Bonferroni.

Conditions: ^1^ Control condition; ^2^ Emsold metatarsal dome 5 mm proximal to metatarsal heads; ^3^ Emsold metatarsal dome in-line with metatarsal heads, ^4^ Emsold metatarsal dome 5 mm distal to metatarsal heads; ^5^ Langer metatarsal dome 5 mm proximal to metatarsal heads; ^6^ Langer metatarsal dome in-line with metatarsal heads, ^7^ Langer metatarsal dome 5 mm distal to metatarsal heads.
