# Supplementary material for: Effects of metatarsal domes on plantar pressures in older people with a history of forefoot pain
Source: J Foot Ankle Res. 2020 May 6;13:18. doi: 10.1186/s13047-020-00388-x (PMC7201604; doi:10.1186/s13047-020-00388-x)
Supplement: Supplementary file 2 — Additional file 2. Pairwise comparisons for mean force (N) at time of peak pressure. [file 13047_2020_388_MOESM2_ESM.docx]

**Additional file 2A** Pairwise comparisons for mean force (N) at time of peak pressure for the proximal mask (N = 36)

| **Condition** | **Comparison** | **Mean difference^*^** | **95% CI**^†^ | | ***P*-value**^†^ |
| --- | --- | --- | --- | --- | --- |
|  | **condition** |  | **Lower limit** | **Upper limit** |  |
| **1** | 2 | -15.398 | -32.902 | 2.106 | 0.141 |
|  | 3 | -16.114^*^ | -31.056 | -1.173 | 0.025 |
|  | 4 | -11.933^*^ | -22.945 | -0.920 | 0.024 |
|  | 5 | -22.070^*^ | -36.596 | -7.545 | <0.001 |
|  | 6 | -18.263 | -39.301 | 2.776 | 0.155 |
|  | 7 | -11.101 | -31.327 | 9.124 | 1.000 |
| **2** | 1 | 15.398 | -2.106 | 32.902 | 0.141 |
|  | 3 | -0.716 | -8.198 | 6.765 | 1.000 |
|  | 4 | 3.465 | -5.943 | 12.874 | 1.000 |
|  | 5 | -6.672 | -19.277 | 5.933 | 1.000 |
|  | 6 | -2.865 | -15.011 | 9.281 | 1.000 |
|  | 7 | 4.297 | -4.487 | 13.081 | 1.000 |
| **3** | 1 | 16.114^*^ | 1.173 | 31.056 | 0.025 |
|  | 2 | 0.716 | -6.765 | 8.198 | 1.000 |
|  | 4 | 4.182 | -5.928 | 14.291 | 1.000 |
|  | 5 | -5.956 | -13.823 | 1.911 | 0.380 |
|  | 6 | -2.148 | -11.123 | 6.826 | 1.000 |
|  | 7 | 5.013 | -4.136 | 14.163 | 1.000 |
| **4** | 1 | 11.933^*^ | 0.920 | 22.945 | 0.024 |
|  | 2 | -3.465 | -12.874 | 5.943 | 1.000 |
|  | 3 | -4.182 | -14.291 | 5.928 | 1.000 |
|  | 5 | -10.138 | -23.476 | 3.200 | 0.371 |
|  | 6 | -6.330 | -23.192 | 10.532 | 1.000 |
|  | 7 | 0.832 | -12.183 | 13.846 | 1.000 |
| **5** | 1 | 22.070^*^ | 7.545 | 36.596 | <0.001 |
|  | 2 | 6.672 | -5.933 | 19.277 | 1.000 |
|  | 3 | 5.956 | -1.911 | 13.823 | 0.380 |
|  | 4 | 10.138 | -3.200 | 23.476 | 0.371 |
|  | 6 | 3.808 | -5.227 | 12.842 | 1.000 |
|  | 7 | 10.969 | -1.565 | 23.504 | 0.146 |
| **6** | 1 | 18.263 | -2.776 | 39.301 | 0.155 |
|  | 2 | 2.865 | -9.281 | 15.011 | 1.000 |
|  | 3 | 2.148 | -6.826 | 11.123 | 1.000 |
|  | 4 | 6.330 | -10.532 | 23.192 | 1.000 |
|  | 5 | -3.808 | -12.842 | 5.227 | 1.000 |
|  | 7 | 7.162 | -1.941 | 16.264 | 0.301 |
| **7** | 1 | 11.101 | -9.124 | 31.327 | 1.000 |
|  | 2 | -4.297 | -13.081 | 4.487 | 1.000 |
|  | 3 | -5.013 | -14.163 | 4.136 | 1.000 |
|  | 4 | -0.832 | -13.846 | 12.183 | 1.000 |
|  | 5 | -10.969 | -23.504 | 1.565 | 0.146 |
|  | 6 | -7.162 | -16.264 | 1.941 | 0.301 |

Notes: Results are based on estimated marginal means. *Mean differences significant at the 0.05 level. ^†^Adjustment for multiple comparisons: Bonferroni.

Conditions: ^1^ Control condition; ^2^ Emsold metatarsal dome 5 mm proximal to metatarsal heads; ^3^ Emsold metatarsal dome in-line with metatarsal heads, ^4^ Emsold metatarsal dome 5 mm distal to metatarsal heads; ^5^ Langer metatarsal dome 5 mm proximal to metatarsal heads; ^6^ Langer metatarsal dome in-line with metatarsal heads, ^7^ Langer metatarsal dome 5 mm distal to metatarsal heads.

**Additional file 2B** Pairwise comparisons for mean force (N) at time of peak pressure for the beneath mask (N = 36)

| **Condition** | **Comparison** | **Mean difference^*^** | **95% CI**^†^ | | ***P*-value**^†^ |
| --- | --- | --- | --- | --- | --- |
|  | **condition** |  | **Lower limit** | **Upper limit** |  |
| **1** | 2 | 17.576^*^ | 2.887 | 32.265 | 0.008 |
|  | 3 | 14.838^*^ | 2.156 | 27.521 | 0.011 |
|  | 4 | 9.852 | -0.789 | 20.493 | 0.095 |
|  | 5 | 13.792^*^ | 2.607 | 24.977 | 0.006 |
|  | 6 | 13.731 | -0.329 | 27.791 | 0.061 |
|  | 7 | 8.445 | -5.886 | 22.776 | 1.000 |
| **2** | 1 | -17.576^*^ | -32.265 | -2.887 | 0.008 |
|  | 3 | -2.738 | -8.613 | 3.138 | 1.000 |
|  | 4 | -7.724^*^ | -15.095 | -0.354 | 0.033 |
|  | 5 | -3.784 | -11.608 | 4.040 | 1.000 |
|  | 6 | -3.845 | -11.114 | 3.424 | 1.000 |
|  | 7 | -9.131^*^ | -16.402 | -1.860 | 0.005 |
| **3** | 1 | -14.838^*^ | -27.521 | -2.156 | 0.011 |
|  | 2 | 2.738 | -3.138 | 8.613 | 1.000 |
|  | 4 | -4.987 | -10.921 | 0.948 | 0.196 |
|  | 5 | -1.047 | -5.768 | 3.675 | 1.000 |
|  | 6 | -1.107 | -6.218 | 4.003 | 1.000 |
|  | 7 | -6.393^*^ | -12.192 | -0.594 | 0.020 |
| **4** | 1 | -9.852 | -20.493 | 0.789 | 0.095 |
|  | 2 | 7.724^*^ | 0.354 | 15.095 | 0.033 |
|  | 3 | 4.987 | -0.948 | 10.921 | 0.196 |
|  | 5 | 3.940 | -3.445 | 11.325 | 1.000 |
|  | 6 | 3.879 | -4.974 | 12.732 | 1.000 |
|  | 7 | -1.407 | -8.924 | 6.111 | 1.000 |
| **5** | 1 | -13.792^*^ | -24.977 | -2.607 | 0.006 |
|  | 2 | 3.784 | -4.040 | 11.608 | 1.000 |
|  | 3 | 1.047 | -3.675 | 5.768 | 1.000 |
|  | 4 | -3.940 | -11.325 | 3.445 | 1.000 |
|  | 6 | -0.061 | -5.400 | 5.278 | 1.000 |
|  | 7 | -5.347 | -11.875 | 1.181 | 0.232 |
| **6** | 1 | -13.731 | -27.791 | 0.329 | 0.061 |
|  | 2 | 3.845 | -3.424 | 11.114 | 1.000 |
|  | 3 | 1.107 | -4.003 | 6.218 | 1.000 |
|  | 4 | -3.879 | -12.732 | 4.974 | 1.000 |
|  | 5 | 0.061 | -5.278 | 5.400 | 1.000 |
|  | 7 | -5.286^*^ | -10.187 | -0.385 | 0.025 |
| **7** | 1 | -8.445 | -22.776 | 5.886 | 1.000 |
|  | 2 | 9.131^*^ | 1.860 | 16.402 | 0.005 |
|  | 3 | 6.393^*^ | 0.594 | 12.192 | 0.020 |
|  | 4 | 1.407 | -6.111 | 8.924 | 1.000 |
|  | 5 | 5.347 | -1.181 | 11.875 | 0.232 |
|  | 6 | 5.286^*^ | 0.385 | 10.187 | 0.025 |

Notes: Results are based on estimated marginal means. *Mean differences significant at the 0.05 level. ^†^Adjustment for multiple comparisons: Bonferroni.

Conditions: ^1^ Control condition; ^2^ Emsold metatarsal dome 5 mm proximal to metatarsal heads; ^3^ Emsold metatarsal dome in-line with metatarsal heads, ^4^ Emsold metatarsal dome 5 mm distal to metatarsal heads; ^5^ Langer metatarsal dome 5 mm proximal to metatarsal heads; ^6^ Langer metatarsal dome in-line with metatarsal heads, ^7^ Langer metatarsal dome 5 mm distal to metatarsal heads.

**Additional file 2C** Pairwise comparisons for mean force (N) at time of peak pressure for the distal mask (N = 36)

| **Condition** | **Comparison** | **Mean difference^*^** | **95% CI**^†^ | | ***P*-value**^†^ |
| --- | --- | --- | --- | --- | --- |
|  | **condition** |  | **Lower limit** | **Upper limit** |  |
| **1** | 2 | 13.016^*^ | 2.962 | 23.069 | 0.003 |
|  | 3 | 13.717^*^ | 6.300 | 21.133 | <0.001 |
|  | 4 | 16.172^*^ | 8.042 | 24.302 | <0.001 |
|  | 5 | 11.670^*^ | 4.415 | 18.926 | <0.001 |
|  | 6 | 16.178^*^ | 8.653 | 23.703 | <0.001 |
|  | 7 | 17.033^*^ | 8.977 | 25.090 | <0.001 |
| **2** | 1 | -13.016^*^ | -23.069 | -2.962 | 0.003 |
|  | 3 | 0.701 | -6.744 | 8.146 | 1.000 |
|  | 4 | 3.156 | -5.013 | 11.326 | 1.000 |
|  | 5 | -1.346 | -9.739 | 7.048 | 1.000 |
|  | 6 | 3.162 | -4.421 | 10.745 | 1.000 |
|  | 7 | 4.017 | -4.979 | 13.014 | 1.000 |
| **3** | 1 | -13.717^*^ | -21.133 | -6.300 | <0.001 |
|  | 2 | -0.701 | -8.146 | 6.744 | 1.000 |
|  | 4 | 2.455 | -1.084 | 5.995 | 0.615 |
|  | 5 | -2.046 | -7.434 | 3.341 | 1.000 |
|  | 6 | 2.461 | -4.088 | 9.011 | 1.000 |
|  | 7 | 3.317 | -2.406 | 9.039 | 1.000 |
| **4** | 1 | -16.172^*^ | -24.302 | -8.042 | <0.001 |
|  | 2 | -3.156 | -11.326 | 5.013 | 1.000 |
|  | 3 | -2.455 | -5.995 | 1.084 | 0.615 |
|  | 5 | -4.502 | -10.703 | 1.699 | 0.483 |
|  | 6 | 0.006 | -8.071 | 8.083 | 1.000 |
|  | 7 | 0.861 | -5.905 | 7.628 | 1.000 |
| **5** | 1 | -11.670^*^ | -18.926 | -4.415 | <0.001 |
|  | 2 | 1.346 | -7.048 | 9.739 | 1.000 |
|  | 3 | 2.046 | -3.341 | 7.434 | 1.000 |
|  | 4 | 4.502 | -1.699 | 10.703 | 0.483 |
|  | 6 | 4.508 | -3.270 | 12.286 | 1.000 |
|  | 7 | 5.363 | -2.886 | 13.612 | 0.847 |
| **6** | 1 | -16.178^*^ | -23.703 | -8.653 | <0.001 |
|  | 2 | -3.162 | -10.745 | 4.421 | 1.000 |
|  | 3 | -2.461 | -9.011 | 4.088 | 1.000 |
|  | 4 | -0.006 | -8.083 | 8.071 | 1.000 |
|  | 5 | -4.508 | -12.286 | 3.270 | 1.000 |
|  | 7 | 0.855 | -5.184 | 6.894 | 1.000 |
| **7** | 1 | -17.033^*^ | -25.090 | -8.977 | <0.001 |
|  | 2 | -4.017 | -13.014 | 4.979 | 1.000 |
|  | 3 | -3.317 | -9.039 | 2.406 | 1.000 |
|  | 4 | -0.861 | -7.628 | 5.905 | 1.000 |
|  | 5 | -5.363 | -13.612 | 2.886 | 0.847 |
|  | 6 | -0.855 | -6.894 | 5.184 | 1.000 |

Notes: Results are based on estimated marginal means. *Mean differences significant at the 0.05 level. ^†^Adjustment for multiple comparisons: Bonferroni.

Conditions: ^1^ Control condition; ^2^ Emsold metatarsal dome 5 mm proximal to metatarsal heads; ^3^ Emsold metatarsal dome in-line with metatarsal heads, ^4^ Emsold metatarsal dome 5 mm distal to metatarsal heads; ^5^ Langer metatarsal dome 5 mm proximal to metatarsal heads; ^6^ Langer metatarsal dome in-line with metatarsal heads, ^7^ Langer metatarsal dome 5 mm distal to metatarsal heads.
