# Supplementary material for: Effects of metatarsal domes on plantar pressures in older people with a history of forefoot pain
Source: J Foot Ankle Res. 2020 May 6;13:18. doi: 10.1186/s13047-020-00388-x (PMC7201604; doi:10.1186/s13047-020-00388-x)
Supplement: Supplementary file 3 — Additional file 3. Pairwise comparisons for contact area (cm2) at time of peak pressure. [file 13047_2020_388_MOESM3_ESM.docx]

**Additional file 3A** Pairwise comparisons for contact area (cm^2^) at time of peak pressure for the proximal mask (N = 36)

| **Condition** | **Comparison** | **Mean difference^*^** | **95% CI**^†^ | | ***P*-value**^†^ |
| --- | --- | --- | --- | --- | --- |
|  | **condition** |  | **Lower limit** | **Upper limit** |  |
| **1** | 2 | -3.580^*^ | -5.028 | -2.132 | <0.001 |
|  | 3 | -3.146^*^ | -4.484 | -1.807 | <0.001 |
|  | 4 | -2.224^*^ | -3.381 | -1.067 | <0.001 |
|  | 5 | -3.697^*^ | -4.921 | -2.472 | <0.001 |
|  | 6 | -2.787^*^ | -4.595 | -0.979 | <0.001 |
|  | 7 | -1.645 | -3.387 | 0.097 | 0.081 |
| **2** | 1 | 3.580^*^ | 2.132 | 5.028 | <0.001 |
|  | 3 | 0.435 | -0.450 | 1.319 | 1.000 |
|  | 4 | 1.356^*^ | 0.411 | 2.301 | 0.001 |
|  | 5 | -0.117 | -0.990 | 0.757 | 1.000 |
|  | 6 | 0.793 | -0.249 | 1.836 | 0.369 |
|  | 7 | 1.935^*^ | 0.759 | 3.110 | <0.001 |
| **3** | 1 | 3.146^*^ | 1.807 | 4.484 | <0.001 |
|  | 2 | -0.435 | -1.319 | 0.450 | 1.000 |
|  | 4 | 0.921 | -0.185 | 2.028 | 0.208 |
|  | 5 | -0.551 | -1.366 | 0.264 | 0.699 |
|  | 6 | 0.359 | -0.674 | 1.392 | 1.000 |
|  | 7 | 1.500^*^ | 0.328 | 2.673 | 0.004 |
| **4** | 1 | 2.224^*^ | 1.067 | 3.381 | <0.001 |
|  | 2 | -1.356^*^ | -2.301 | -0.411 | 0.001 |
|  | 3 | -0.921 | -2.028 | 0.185 | 0.208 |
|  | 5 | -1.473^*^ | -2.549 | -0.397 | 0.002 |
|  | 6 | -0.563 | -2.138 | 1.013 | 1.000 |
|  | 7 | 0.579 | -0.849 | 2.006 | 1.000 |
| **5** | 1 | 3.697^*^ | 2.472 | 4.921 | <0.001 |
|  | 2 | 0.117 | -0.757 | 0.990 | 1.000 |
|  | 3 | 0.551 | -0.264 | 1.366 | 0.699 |
|  | 4 | 1.473^*^ | 0.397 | 2.549 | 0.002 |
|  | 6 | 0.910 | -0.221 | 2.041 | 0.261 |
|  | 7 | 2.051^*^ | 0.648 | 3.454 | 0.001 |
| **6** | 1 | 2.787^*^ | 0.979 | 4.595 | <0.001 |
|  | 2 | -0.793 | -1.836 | 0.249 | 0.369 |
|  | 3 | -0.359 | -1.392 | 0.674 | 1.000 |
|  | 4 | 0.563 | -1.013 | 2.138 | 1.000 |
|  | 5 | -0.910 | -2.041 | 0.221 | 0.261 |
|  | 7 | 1.141^*^ | 0.118 | 2.165 | 0.018 |
| **7** | 1 | 1.645 | -0.097 | 3.387 | 0.081 |
|  | 2 | -1.935^*^ | -3.110 | -0.759 | <0.001 |
|  | 3 | -1.500^*^ | -2.673 | -0.328 | 0.004 |
|  | 4 | -0.579 | -2.006 | 0.849 | 1.000 |
|  | 5 | -2.051^*^ | -3.454 | -0.648 | 0.001 |
|  | 6 | -1.141^*^ | -2.165 | -0.118 | 0.018 |

Notes: Results are based on estimated marginal means. *Mean differences significant at the 0.05 level. ^†^Adjustment for multiple comparisons: Bonferroni.

Conditions: ^1^ Control condition; ^2^ Emsold metatarsal dome 5 mm proximal to metatarsal heads; ^3^ Emsold metatarsal dome in-line with metatarsal heads, ^4^ Emsold metatarsal dome 5 mm distal to metatarsal heads; ^5^ Langer metatarsal dome 5 mm proximal to metatarsal heads; ^6^ Langer metatarsal dome in-line with metatarsal heads, ^7^ Langer metatarsal dome 5 mm distal to metatarsal heads.

**Additional file 3B** Pairwise comparisons for contact area (cm^2^) at time of peak pressure for the beneath mask (N = 36)

| **Condition** | **Comparison** | **Mean difference^*^** | **95% CI**^†^ | | ***P*-value**^†^ |
| --- | --- | --- | --- | --- | --- |
|  | **condition** |  | **Lower limit** | **Upper limit** |  |
| **1** | 2 | 0.110 | -0.066 | 0.286 | 1.000 |
|  | 3 | 0.092 | -0.101 | 0.284 | 1.000 |
|  | 4 | 0.094 | -0.124 | 0.312 | 1.000 |
|  | 5 | 0.110 | -0.088 | 0.308 | 1.000 |
|  | 6 | 0.191 | -0.051 | 0.434 | 0.293 |
|  | 7 | 0.079 | -0.074 | 0.232 | 1.000 |
| **2** | 1 | -0.110 | -0.286 | 0.066 | 1.000 |
|  | 3 | -0.018 | -0.227 | 0.190 | 1.000 |
|  | 4 | -0.016 | -0.180 | 0.148 | 1.000 |
|  | 5 | 0.000 | -0.158 | 0.158 | 1.000 |
|  | 6 | 0.081 | -0.136 | 0.298 | 1.000 |
|  | 7 | -0.031 | -0.189 | 0.127 | 1.000 |
| **3** | 1 | -0.092 | -0.284 | 0.101 | 1.000 |
|  | 2 | 0.018 | -0.190 | 0.227 | 1.000 |
|  | 4 | 0.002 | -0.187 | 0.192 | 1.000 |
|  | 5 | 0.018 | -0.118 | 0.155 | 1.000 |
|  | 6 | 0.100 | -0.126 | 0.325 | 1.000 |
|  | 7 | -0.012 | -0.169 | 0.144 | 1.000 |
| **4** | 1 | -0.094 | -0.312 | 0.124 | 1.000 |
|  | 2 | 0.016 | -0.148 | 0.180 | 1.000 |
|  | 3 | -0.002 | -0.192 | 0.187 | 1.000 |
|  | 5 | 0.016 | -0.148 | 0.180 | 1.000 |
|  | 6 | 0.098 | -0.127 | 0.322 | 1.000 |
|  | 7 | -0.014 | -0.197 | 0.168 | 1.000 |
| **5** | 1 | -0.110 | -0.308 | 0.088 | 1.000 |
|  | 2 | 0.000 | -0.158 | 0.158 | 1.000 |
|  | 3 | -0.018 | -0.155 | 0.118 | 1.000 |
|  | 4 | -0.016 | -0.180 | 0.148 | 1.000 |
|  | 6 | 0.081 | -0.126 | 0.288 | 1.000 |
|  | 7 | -0.031 | -0.224 | 0.162 | 1.000 |
| **6** | 1 | -0.191 | -0.434 | 0.051 | 0.293 |
|  | 2 | -0.081 | -0.298 | 0.136 | 1.000 |
|  | 3 | -0.100 | -0.325 | 0.126 | 1.000 |
|  | 4 | -0.098 | -0.322 | 0.127 | 1.000 |
|  | 5 | -0.081 | -0.288 | 0.126 | 1.000 |
|  | 7 | -0.112 | -0.340 | 0.115 | 1.000 |
| **7** | 1 | -0.079 | -0.232 | 0.074 | 1.000 |
|  | 2 | 0.031 | -0.127 | 0.189 | 1.000 |
|  | 3 | 0.012 | -0.144 | 0.169 | 1.000 |
|  | 4 | 0.014 | -0.168 | 0.197 | 1.000 |
|  | 5 | 0.031 | -0.162 | 0.224 | 1.000 |
|  | 6 | 0.112 | -0.115 | 0.340 | 1.000 |

Notes: Results are based on estimated marginal means. *Mean differences significant at the 0.05 level. ^†^Adjustment for multiple comparisons: Bonferroni.

Conditions: ^1^ Control condition; ^2^ Emsold metatarsal dome 5 mm proximal to metatarsal heads; ^3^ Emsold metatarsal dome in-line with metatarsal heads, ^4^ Emsold metatarsal dome 5 mm distal to metatarsal heads; ^5^ Langer metatarsal dome 5 mm proximal to metatarsal heads; ^6^ Langer metatarsal dome in-line with metatarsal heads, ^7^ Langer metatarsal dome 5 mm distal to metatarsal heads.

**Additional file 3C** Pairwise comparisons for contact area (cm^2^) at time of peak pressure for the distal mask (N = 36)

| **Condition** | **Comparison** | **Mean difference^*^** | **95% CI**^†^ | | ***P*-value**^†^ |
| --- | --- | --- | --- | --- | --- |
|  | **condition** |  | **Lower limit** | **Upper limit** |  |
| **1** | 2 | 0.071 | -0.059 | 0.201 | 1.000 |
|  | 3 | 0.071 | -0.060 | 0.202 | 1.000 |
|  | 4 | 0.043 | -0.057 | 0.142 | 1.000 |
|  | 5 | 0.000 | 0.000 | 0.000 | 1.000 |
|  | 6 | 0.038 | -0.049 | 0.124 | 1.000 |
|  | 7 | 0.062 | -0.054 | 0.178 | 1.000 |
| **2** | 1 | -0.071 | -0.201 | 0.059 | 1.000 |
|  | 3 | 0.001 | -0.105 | 0.106 | 1.000 |
|  | 4 | -0.028 | -0.146 | 0.090 | 1.000 |
|  | 5 | -0.071 | -0.201 | 0.059 | 1.000 |
|  | 6 | -0.033 | -0.194 | 0.128 | 1.000 |
|  | 7 | -0.009 | -0.144 | 0.127 | 1.000 |
| **3** | 1 | -0.071 | -0.202 | 0.060 | 1.000 |
|  | 2 | -0.001 | -0.106 | 0.105 | 1.000 |
|  | 4 | -0.029 | -0.148 | 0.091 | 1.000 |
|  | 5 | -0.071 | -0.202 | 0.060 | 1.000 |
|  | 6 | -0.034 | -0.196 | 0.129 | 1.000 |
|  | 7 | -0.009 | -0.146 | 0.128 | 1.000 |
| **4** | 1 | -0.043 | -0.142 | 0.057 | 1.000 |
|  | 2 | 0.028 | -0.090 | 0.146 | 1.000 |
|  | 3 | 0.029 | -0.091 | 0.148 | 1.000 |
|  | 5 | -0.043 | -0.142 | 0.057 | 1.000 |
|  | 6 | -0.005 | -0.115 | 0.105 | 1.000 |
|  | 7 | 0.020 | -0.044 | 0.083 | 1.000 |
| **5** | 1 | 0.000 | 0.000 | 0.000 | 1.000 |
|  | 2 | 0.071 | -0.059 | 0.201 | 1.000 |
|  | 3 | 0.071 | -0.060 | 0.202 | 1.000 |
|  | 4 | 0.043 | -0.057 | 0.142 | 1.000 |
|  | 6 | 0.038 | -0.049 | 0.124 | 1.000 |
|  | 7 | 0.062 | -0.054 | 0.178 | 1.000 |
| **6** | 1 | -0.038 | -0.124 | 0.049 | 1.000 |
|  | 2 | 0.033 | -0.128 | 0.194 | 1.000 |
|  | 3 | 0.034 | -0.129 | 0.196 | 1.000 |
|  | 4 | 0.005 | -0.105 | 0.115 | 1.000 |
|  | 5 | -0.038 | -0.124 | 0.049 | 1.000 |
|  | 7 | 0.024 | -0.103 | 0.152 | 1.000 |
| **7** | 1 | -0.062 | -0.178 | 0.054 | 1.000 |
|  | 2 | 0.009 | -0.127 | 0.144 | 1.000 |
|  | 3 | 0.009 | -0.128 | 0.146 | 1.000 |
|  | 4 | -0.020 | -0.083 | 0.044 | 1.000 |
|  | 5 | -0.062 | -0.178 | 0.054 | 1.000 |
|  | 6 | -0.024 | -0.152 | 0.103 | 1.000 |

Notes: Results are based on estimated marginal means. *Mean differences significant at the 0.05 level. ^†^Adjustment for multiple comparisons: Bonferroni.

Conditions: ^1^ Control condition; ^2^ Emsold metatarsal dome 5 mm proximal to metatarsal heads; ^3^ Emsold metatarsal dome in-line with metatarsal heads, ^4^ Emsold metatarsal dome 5 mm distal to metatarsal heads; ^5^ Langer metatarsal dome 5 mm proximal to metatarsal heads; ^6^ Langer metatarsal dome in-line with metatarsal heads, ^7^ Langer metatarsal dome 5 mm distal to metatarsal heads.
